# Re-Identification Risk versus Data Utility for Aggregated Mobility Research Using Mobile Phone Location Data

**DOI:** 10.1371/journal.pone.0140589

**Published:** 2015-10-15

**Authors:** Ling Yin, Qian Wang, Shih-Lung Shaw, Zhixiang Fang, Jinxing Hu, Ye Tao, Wei Wang

**Affiliations:** 1 Shenzhen Institutes of Advanced Technology, Chinese Academy of Sciences, Shenzhen, Guangdong, China; 2 Department of Geography, The University of Tennessee, Knoxville, TN, United States of America; 3 State Key Laboratory of Information Engineering in Surveying, Mapping, and Remote Sensing, Wuhan University, Wuhan, Hubei, China; 4 Department of Urban Planning and Design, The University of Hong Kong, Hong Kong, China; East China University of Science and Technology, CHINA

## Abstract

Mobile phone location data is a newly emerging data source of great potential to support human mobility research. However, recent studies have indicated that many users can be easily re-identified based on their unique activity patterns. Privacy protection procedures will usually change the original data and cause a loss of data utility for analysis purposes. Therefore, the need for detailed data for activity analysis while avoiding potential privacy risks presents a challenge. The aim of this study is to reveal the re-identification risks from a Chinese city’s mobile users and to examine the quantitative relationship between re-identification risk and data utility for an aggregated mobility analysis. The first step is to apply two reported attack models, the top *N* locations and the spatio-temporal points, to evaluate the re-identification risks in Shenzhen City, a metropolis in China. A spatial generalization approach to protecting privacy is then proposed and implemented, and spatially aggregated analysis is used to assess the loss of data utility after privacy protection. The results demonstrate that the re-identification risks in Shenzhen City are clearly different from those in regions reported in Western countries, which prove the spatial heterogeneity of re-identification risks in mobile phone location data. A uniform mathematical relationship has also been found between re-identification risk (*x*) and data (*y*) utility for both attack models: *y* = -*ax* ^*b*^+*c*, (*a*, *b*, *c*>0; 0<*x*<1), where the exponent b increases with the background knowledge of the attackers. The discovered mathematical relationship provides data publishers with useful guidance on choosing the right tradeoff between privacy and utility. Overall, this study contributes to a better understanding of re-identification risks and a privacy-utility tradeoff benchmark for improving privacy protection when sharing detailed trajectory data.

## Introduction

As a routine procedure, mobile phone operators collect users’ location data with certain sampling methods for billing, troubleshooting, or other technical measurement purposes. The ability to obtain massive numbers of individual trajectories automatically and remotely opens up unprecedented opportunities to study human mobility with a large sample size at low cost [1]. With mobile phone location data, a number of studies over the past few years have made progress in formulating universal human mobility patterns [2–4], predicting human mobility [5–6], estimating origin-destination (OD) flows [7–9], modeling human movement [10], revealing population dynamics and hot spots [11–12], identifying important activity places [13], and mining daily activity structures [14]. These studies have suggested that mobile phone location data have the potential to become a major data source to study human mobility for transportation, urban planning, epidemiology, and sociology research.

However, the rich spatio-temporal information embedded in such trajectory data may result in privacy breach. Two recent studies have pointed out that from anonymized mobile phone location datasets, and with only a little outside information, the uniqueness of individual trajectories can cause identity exposure (re-identification) for a considerable number of mobile users. The MobiCom 2011 study proposed an attack model based on the top *N* locations and demonstrated that at the spatial granularity of the mobile sector or mobile cell level, the top two or three locations from mobile users’ trajectories yielded unique identifications of 10%–50% of individuals in the United States [15]. The Scientific Report 2013 study proposed an attack model based on spatial-temporal points and revealed that four randomly selected spatio-temporal points could uniquely identify 95% of the mobile users in a European country [16].

The severe re-identification risks revealed by these studies and the increasing number of studies using mobile phone location data impel the community to engage in a deeper discussion about the right way to safeguard privacy when working with these kinds of data [17]. On the one hand, researchers and policy-makers would like to have detailed trajectory data to learn important information that would benefit society; on the other hand, data publishers must apply privacy protection procedures on original data to make sure that sharing the data does not lead to privacy breach. Researchers have proposed some approaches to protecting trajectory privacy [18–21], such as adding dummy data [22] or suppressing or generalizing sensitive information [23–27]. However, existing research on other datasets has suggested that the more privacy we protect, the more data utility will be lost [28–32]. Generally, it is challenging to find an optimal tradeoff between these two conflicting goals. In terms of data utility for human mobility research, some studies have aimed to analyze the mobility patterns of a particular individual, such as identifying activity locations with a spatial resolution of hundreds of meters [13–14]. In this case, the changes to individual traces introduced by a privacy protection procedure such as location perturbation are likely to have a significant impact on the results of the analysis. On the other hand, many policy-making applications focus only on aggregated mobility patterns such as OD flows between regions with a spatial resolution of thousands of meters, a fact which likely enlarges the space for compromise between privacy protection and data utility.

To deepen our understanding of the privacy risks in mobile phone location data and to help develop useful privacy protection approaches when sharing such data, this study uses an anonymized mobile phone location dataset from a major city in China to address three issues. First, do the high re-identification risks in mobile phone location datasets from the United States [15] and a European country [16] also apply to a major city in China that has a different population size, culture, and lifestyle? To the best of our knowledge, a comparative study across countries has not yet been published. Second, how do generalization-based approaches, as suggested in [15] and [16], reduce re-identification risk and affect human mobility analysis, especially at the aggregated analysis level of policy-making? Third, this study seeks to explore the possibility of a quantitative relationship between re-identification risk and data utility for aggregated mobility analysis. If there is such a relationship, what is the math formula? Answers to these questions could help to estimate re-identification risks in different regions and to understand the tradeoff between privacy risk and data utility, thus making it possible to design better privacy protection approaches and to improve sustainable use of mobile phone location data.

## Materials and Methods

### Dataset

Because previous studies [15] and [16] conducted their privacy risk measurements based on call detail records (CDR), which are a common type of mobile phone location dataset, the same data type was chosen for this study to enable rigorous comparison of results. The local transportation department provided the CDR dataset and permitted this research and publication of its results. Each CDR contains the location and time when a user initiates or receives a call, thus providing an individual trajectory data source. The CDR dataset used in this study was collected by a major telecom operator in Shenzhen City, a metropolis with the highest population density and fourth highest economic output among Chinese cities (http://english.sz.gov.cn/). Shenzhen City covers an area of 1952 km^2^, where there are 3036 base stations owned by the telecom operator. This CDR dataset includes call records of 4.57 million mobile users in 2011 (nearly 30% of the total population) and covers a span of 34 days, including 25 weekdays and 9 weekend days. Each entry in the dataset is made up of six attribute fields (S1 Table): (*ID*) the user’s ID (encrypted by the operator), (*t*) time when the call was connected, (*lat*, *long*) latitude and longitude of the user’s connected base station, (*f*) a flag representing whether the call was initiated or received, and (*reg*) the regional phone code. This dataset itself uses three methods to protect privacy: encrypting users’ mobile phone numbers; generalizing every user’s precise location with a base station coverage area; and inhibiting exposure of user trajectories through sparse location sampling only triggered by call events.

### Re-identification Risk Evaluation

This study defines individual privacy risk as the probability of being re-identified. To compare privacy risks in different geographical regions, the same methods proposed in [15] and [16] were used to evaluate the privacy risk in this dataset. These methods include two attack models using the top *N* locations [15] and spatio-temporal points [16] as quasi-identifiers.

The top *N* locations refer to the locations observed to be most frequently visited by a mobile user. This model assumes that attackers have background knowledge of a target’s top *N* locations. In reality, the top *N* locations are usually important places for residents. For instance, the top two locations likely correspond to a user’s home and work places, and the third top location is likely to be a kindergarten, school, or shopping mall that a user often visits. In today’s information society, it is not difficult for attackers to obtain such outside knowledge. Therefore, the attack scenario using the top *N* locations is reasonable and generally applicable. Specifically, a person’s re-identification risk is quantified by the *k-anonymity value* (*k*∈N*), of which *k* represents the size of an anonymity set that includes users with the same top *N* locations [24]. The re-identification risk decreases with the size of the anonymity set *k*. In the extreme case where *k* equals 1 (i.e., there is only one user in the anonymity set), the re-identification risk of this user reaches 100%. The top *N* location model also has another two extended forms: the unordered top *N* locations, and the variable-length top locations. The unordered top *N* location model treats top locations identically regardless of their order. The variable-length top location model calculates the number of “preferred” locations for each user, and the number of top locations *N* varies for each user. For details of these two extended forms, interested readers can refer to [15]. Besides, the top *N* location model only works for frequent call users. For mobile users who made or received very few calls during the observation period, it is difficult to infer their top *N* locations accurately. Study [15] defines frequent call users as mobile users who made or received at least one call per day in average.

The second attack model represents a user *U*’s trace by *Trace*_U_ = {*lat*_1_-*long*_1_-*t*_1_, *lat*_2_-*long*_2_-*t*_2_, *lat*_3_-*long*_3_-*t*_3_,…… *lat*_n_-*long*_n_-*t*_n_}, where the spatio-temporal point (*lat*_i_-*long*_i_-*t*_i_) represents the location of *U* at time *t*_i_ and *n* is the number of observed spatio-temporal points in the trajectory. This model supposes that attackers know *p* spatio-temporal points of the target and simulates this uncertain background knowledge by randomly selecting *p* points. This attack assumption also has wide applicability, especially with the extensive use of mobile social media such as Twitter that can be used to acquire self-reported whereabouts. The *k*-anonymity method is also used to quantify privacy risks. In this case, *k* represents the size of the anonymity set composed of users with the same selected spatio-temporal points. Similarly, users in an anonymity set with size *k* equals to one can be 100% re-identified. This spatio-temporal point model works for all mobile users.

A uniquely identified population ratio *x* (*x* = population with *k* equal to 1/total population) has been suggested as a useful indicator to represent the re-identification risk in a population [16]. This study therefore uses this identified population ratio *x* to evaluate the impact of privacy protection on privacy risk reduction and to formulate the relationship between privacy risk and data utility.

### Privacy Protection Procedure

**Fig 1:**
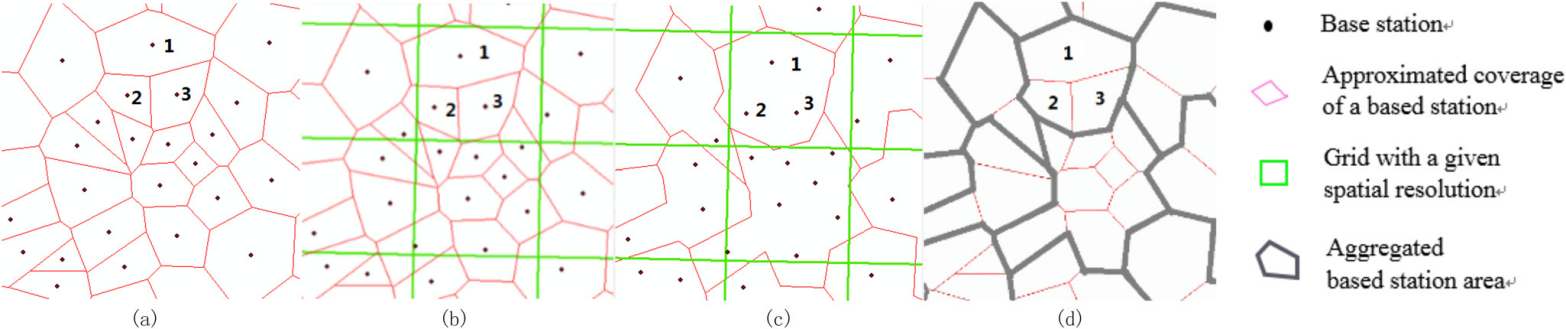
Privacy protection method for mobile phone location dataset by aggregating base stations. (a) Original base stations. (b) Grid creation. (c) Base station aggregation. (d) Aggregation results.

This study proposes a privacy protection method which reduces the spatial precision of a user’s location, which is a generalization-based approach. As Fig 1a shows, a base station coverage area can be represented by a Voronoi polygon. Many factors can influence the coverage area, including its surrounding topography and buildings and the height and angle of its antennas. If only the locations of base stations are available, the best approximation is to use Voronoi polygons [2,5,16]. Then a grid with a given spatial aggregation resolution is created for the study area (Fig 1b). Base stations with centroids located in the same grid block are aggregated (Fig 1c), and the aggregated base station is treated as a new spatial unit for data publication (Fig 1d). For instance, as shown in Fig 1, an original CDR record at base station 2 (*lat*_2_, *long*_2_, *t*) will be changed to ({(*lat*_1_, *long*_1_), (*lat*_2_, *long*_2_), (*lat*_3_, *long*_3_)}, *t*). With a larger location range, mobile users will more likely to co-locate with others, and their trace uniqueness can be expect to decrease.

To measure the impact of spatial aggregation resolution on privacy protection systematically, the base stations were aggregated into a series of grids ranging from 200 m × 200 m to 2800 m × 2800 m with an increment of 200 m. S1 Fig shows the results of this aggregation.

### Data Utility Evaluation

Because an OD flow matrix is a basic input for many transportation models and is of prime importance for studies based on human mobility, generation of an OD flow matrix was chosen here as a representative kind of aggregated analysis among various data utilities for mobile phone location data.

To measure data utility loss, the differences between two OD flow matrices derived from the dataset before and after privacy protection were assessed. Then data utility loss was calculated as the percentage of the total flow difference over the total flow during a time period *T* (Eq 1):

$$utility\;loss=\frac{\sum_{i=1}^{N}\sum_{j=1}^{N}\left|OD_{ij}-OD\prime_{ij}\right|}{\sum_{i=1}^{N}\sum_{j=1}^{N}OD_{ij}}$$ (1)

where OD_ij_ is the original flux from region *i* to region *j* during *T*, OD′_ij_ is the flux from region *i* to region *j* during *T* after privacy protection, and *N* is the number of regions.

As the regional unit in Eq 1, traffic analysis zones (TAZs) were chosen. TAZs are designed for mobility studies, usually by planning practitioners based on regional socioeconomic characteristics, and many transportation models require TAZ-based OD flow matrices OD^TAZ^ as inputs. TAZs may have different spatial resolutions according to application requirements. To compare the impacts on data utility of different spatial resolutions, this study used two sets of TAZs, one having 1112 TAZs with an average radius of 636 meters, and the other having 491 TAZs with an average radius of 994 meters. The radius of a TAZ is measured by the radius of a circle with the same area. The two sets of TAZs are provided by *City Urban Planning*, *Land & Resources Commission of Shenzhen Municipality* (http://www.szpl.gov.cn/). To represent the impact on whole dataset, the time period *T* is set as the entire time span of the CDR dataset (i.e., 34 days).

For the OD flow matrix before privacy protection, the first step was to apply the concept of transient-origin-destination [12] to identify movements between base stations. This method identifies a movement by searching for two consecutive non-identical locations from a trajectory and sets the two locations as origin and destination respectively according to their temporal order. This study named these OD flows as *All flows-Raw*, which represent the mobility patterns inherent in a CDR dataset. Because call events have been proved to be non-uniformly distributed over time [33] and a CDR dataset contains only a user’s locations when a call event happens, the CDR is sparsely sampled, and this sampling method greatly influences the accuracy of estimating OD flows. For instance, a person may make a first call at place A in the morning, then make several trips without any calls, and finally make a second call at place B in the evening. The OD A→B derived from her/his CDR obviously provides wrong travel information for mobility studies. Moreover, in mobile networks, signal transitions between neighboring base stations will produce false short-distance movements [14]. To reduce these two types of errors in *All flows-Raw*, travel time of a single trip was used as a constraint to refine the identified movements. Based on a travel dairy of Shenzhen compiled only six months before the collection date of the mobile dataset used in this study, 98% of trips have a travel time between 5 minutes and 100 minutes (as shown in S2 Fig). This study excluded the movements with time intervals less than 5 minutes or greater than 100 minutes, and named the remaining OD flows as *All flows-Constraint*. Then the spatial unit of the OD flow matrix was converted from base stations to TAZs. The flow $OD_{ij}^{TAZ}$ from TAZ *i* to TAZ *j* is generated by Eq 2:

$$OD_{ij}^{TAZ}=\sum_{k=1}^{M}\sum_{l=1}^{M}\frac{A_{i}^{k}}{A^{k}}\cdot \frac{A_{j}^{l}}{A^{l}}\cdot OD_{kl}^{BS}$$ (2)

where base station *k* with area A^*k*^ has an overlapping area A^*k*^_*i*_ with TAZ *i*, base station *l* with area A^*l*^ has an overlapping area A^*l*^_*j*_ with TAZ *j*, OD^*BS*^_*kl*_ is the flux from base station *k* to base station *l*, and *M* is the number of base stations.

After privacy protection, suppose there are *S* aggregated base stations. The first step is to create flow OD^*AgrBS*^_*pq*_ from aggregated base station *p* to *q* using Eq 3. Then Eq 4 is used to generate the TAZ-based OD′^*TAZ*^_*ij*_ after privacy protection. Finally, Eq 1 is used to calculate the utility loss:

$$OD_{pq}^{AgrBS}=\sum_{u=1}^{U}\sum_{v=1}^{V}OD_{uv}^{BS}$$ (3)

where aggregated base station *p* includes *U* base stations, aggregated base station *q* includes *V* base stations, and OD^*BS*^_*uv*_ is the flux from base station *u* to base station *v*:

$$OD\prime_{ij}^{TAZ}=\sum_{p=1}^{S}\sum_{q=1}^{S}\frac{A_{i}^{p}}{A^{p}}\cdot \frac{A_{j}^{q}}{A^{q}}\cdot OD_{pq}^{AgrBS}$$ (4)

where aggregated base station *p* with area A^*p*^ has an overlapping area A^*p*^_*i*_ with TAZ *i*, and aggregated base station *q* with area A^*q*^ has an overlapping area A^q^_*j*_ with TAZ *j*.

Moreover, the *Pareto principle* (also known as the *80–20 rule*) broadly exists in transportation systems. This principle states that roughly 20% of road segments (i.e., major roads) carry 80% of the total traffic flow. The Pareto rule suggests that a small proportion of OD pairs contributes most of the flows, and these OD pairs are more important than the remaining ones in a transportation system. Obviously, there exist some major flows that received impact from privacy protection procedures should be particularly examined in the results analysis. Because the definition of major flows can be various and may affect our analysis results, this study adopted three ways to define major flows. The first one was designed simply according to the Pareto principle. This study ranked OD pairs by their flux from high to low and selected the top 80% of flows as major flows, named *Major flows-Pareto*. We found that the top 80% of flows was contributed by less than 2% of OD pairs (see S3 Fig for details), which is an extreme example of the Pareto principle. The second definition of major flows followed the network’s backbone proposed by the study [34]. It filters a network’s backbone from a weighted network by considering local heterogeneity and local correlations among relevant edges, so that it does not belittle small-scale interactions. This study names the backbone of networks as *Major flows-Backbone*. The third one defines major flows as the flows that start from or end with hotspots based on the study [35]. This study defined the hotspots as TAZs whose population are higher than a threshold. We estimated the population of a TAZ by summing up hourly caller volume in this TAZ through the study period. The population threshold is derived from the Lorenz curve of the population distribution [36]. This study names the flows selected by the third definition as *Major flows-Hotspot*. For details of the second and third definitions, interested readers can refer to [34–36]. This study calculated the above three types of major flows respectively based on All flows-Raw and All flows-Constraint, and labeled them with ‘-Raw’ and–‘Constraint’ in the results analysis.

## Results and Discussion

### Re-identification Risk

**Fig 2:**
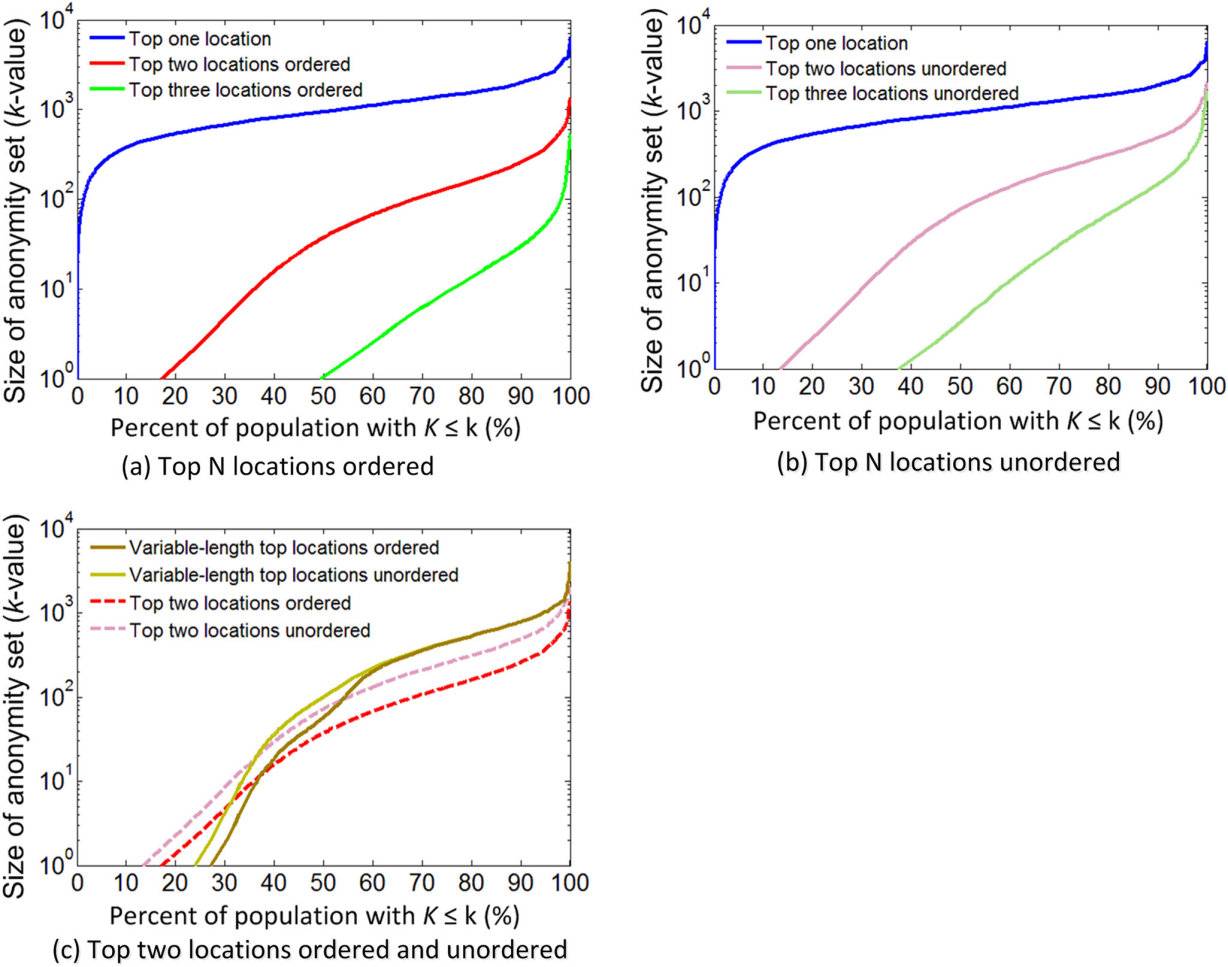
Size of anonymity set for the top N locations. (a) Top N locations ordered. (b) Top N locations unordered. (c) Top two locations ordered and unordered.

For the attack model using the top *N* locations, the size of the anonymity set (*k*-anonymity value) was calculated for each frequent call user, and the *k*-anonymity value was plotted for the proportion of users with *K≤k* (Fig 2). The three curves representing the top one, two, and three locations show that the *k*-anonymity value in general decreases with increasing number of top locations, which demonstrates the increased re-identification risk with additional background knowledge about top locations. As illustrated in Fig 2a and Table 1 (see “For frequent call users”), almost no users are uniquely identified by their top one location, which means that if only the most frequently visited location is exposed, there is almost nothing to worry about. However, when the top two and top three locations are revealed, the uniquely identified population increases to **17%** and **49%** respectively. Fig 2b shows the *k*-anonymity values for the top one, two, and three locations unordered, and Fig 2c compares the two curves for the ordered and unordered top two locations. The re-identification risk from the unordered top two locations is nearly 5% less than that from the ordered top two locations. In other words, the additional background knowledge about the order of the top two locations increases the re-identification risk for users by approximately 5%. Fig 2c also shows the cases using the variable-length top locations ordered and unordered. The two curves are close to the curves of the top two locations. When the *k*-anonymity value is smaller than 10–20, the curves of the variable-length top locations are located below the curves of the top two locations. As the size of the anonymity set increases, the two curves of the variable-length top locations move higher than the curves of the top two locations, and gradually the two curves become identical. These results indicate that most users with relatively high re-identification risks (higher than 1/20–1/10) have only two preferred locations, and most users with relatively low re-identification risks have only one preferred location.

**Table 1:** Percentage of population with *k*-anonymity value = 1 in Shenzhen City and the United States.

User group	Region	Top 1 location (%)	Top 2 locations (%)	Top 3 locations (%)
**Frequent call users**	**Shenzhen**	~0	17	49
	**United States** *	~0	15	50
**All mobile users**	**Shenzhen**	~0	6	18
	**United States** *	~0	12	40

* The results in the United States for frequent users are reported in [15]. The results in the United States for all users are not reported in [15], but were calculated by the authors of this study based on the reported percentage of users with *k* = 1 among frequent users and the percentage of frequent call users among all users.

The *k*-anonymity values for the 1^st^, 5^th^, 10^th^, and the 50^th^ percentiles of frequent call users were then compared in the results of this study and those for the entire United States as reported in [15] (Table 2). Shenzhen City had a re-identification risk fairly similar to that of the overall United States in terms of frequent call users. Without results from other regions in the world, the authors currently consider this consistency to be coincidental.

**Table 2:** *k*-anonymity value with top *N* locations (*N* = 1, 2, 3) in Shenzhen City and the United States.

User group	Region	Top 1 location (%ile)				Top 2 locations (%ile)				Top 3 locations (%ile)			
		1^st^	5^th^	10^th^	50^th^	1^st^	5^th^	10^th^	50^th^	1^st^	5^th^	10^th^	50^th^
**Frequent call users**	**Shenzhen**	82	246	376	943	1	1	1	38	1	1	1	1
	**United States**	92	220	331	967	1	1	1	9	1	1	1	1

* The results in the United States for frequent users are reported in [15].

The percentage of frequent call users with *k*-anonymity value = 1 was also compared in the results of this research and in results from various cities in the United States [15] (Table 3). The percentage of frequent users with *k*-anonymity value = 1 in Shenzhen is between that of Sacramento and that of Chicago. Study [15] suggests that re-identification risk might be related to the differences between urban and rural lifestyles. A more urban lifestyle causes more diversified activities, thus generating smaller anonymity sets. According to this explanation, the degree of urban lifestyle in Shenzhen might be between that of Sacramento and that of Chicago.

**Table 3:** Percentage of frequent users with *k*-anonymity value = 1 in different cities (*N* = 2).

Cities	Shenzhen (China)	Kansas City*(U.S.)	Sacramento*(U.S.)	Chicago*(U.S.)	Los Angeles*(U.S.)	San Francisco*(U.S.)
**Percent of frequent users with *k*-anonymity value = 1 (%)**	17	10	15	25	25	30
**Population density (persons per mi** ^**2**^ **)**	20,205	1,168	4,660	11,868	12,451	17,246

* The percentages of the population with *k* = 1 in various cities in the United States are reported in [15]. The population densities of cities in the United States were estimated based on the 2010 United States census results. They might vary according to the definition of city boundaries.

These results and discussions are based on frequent call users. If non-frequent call users are treated as a population with no re-identification risk and the re-identification risk is calculated for all mobile users, the uniquely identified population in Shenzhen City is only half that in the United States for the top two locations and less than half for the top three locations (see “For all mobile users” in Table 1). Shenzhen City has only 37% frequent call users among its mobile users, whereas United States has 80% frequent call users. The significant higher ratio of inactivity mobile users in Shenzhen City could be related with several situations, such as mobile users in Shenzhen City make much fewer calls than in the United States, many Shenzhen mobile users use more than one telephone number or some telephone numbers have been left nearly unused. One or several of the above possible reasons lead to lower trajectory exposure and lower overall privacy risk for mobile users in Shenzhen City than in the United States.

**Fig 3:**
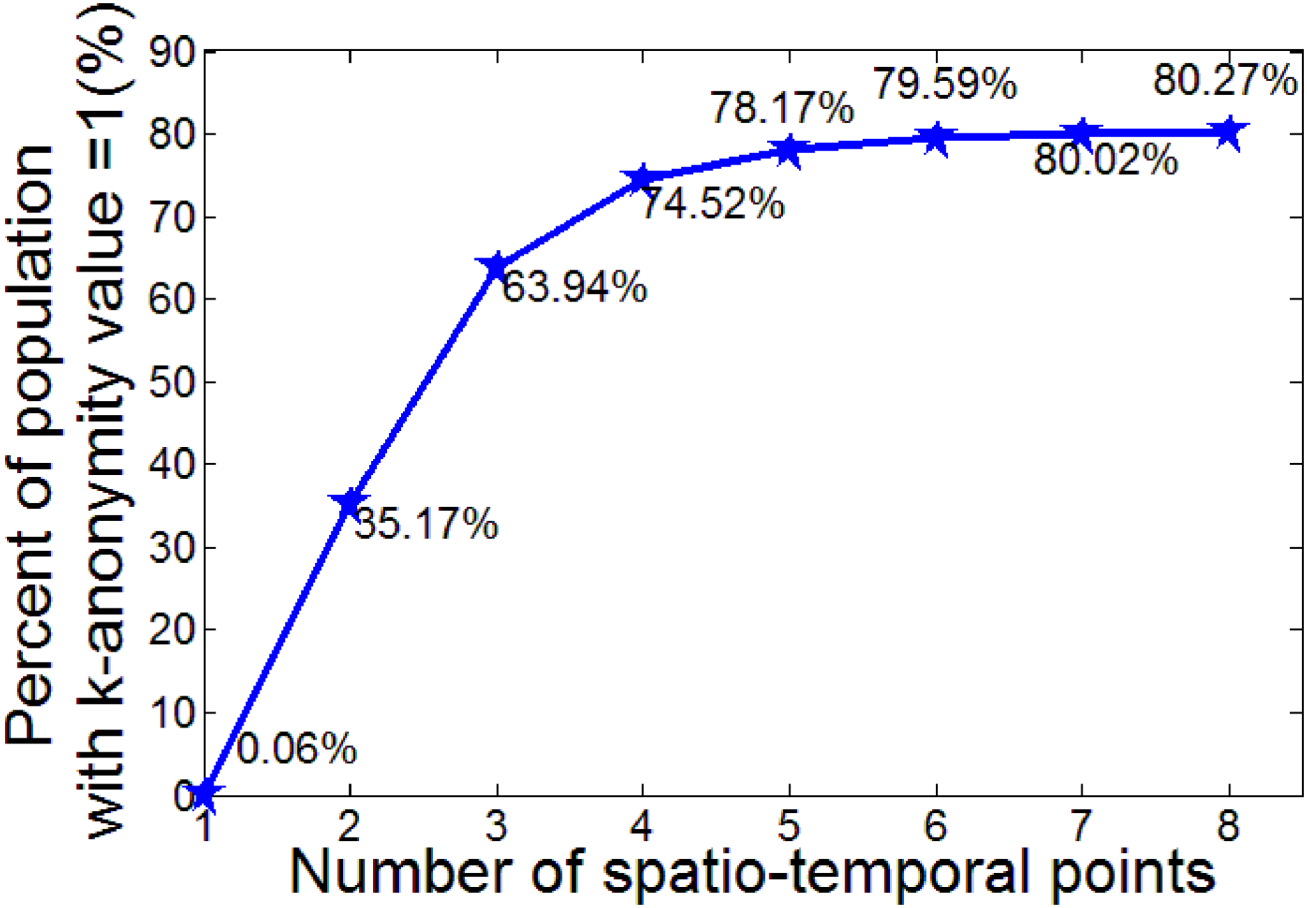
Population with 100% re-identification risk under different number of spatio-temporal points.

In the attack model using spatio-temporal points, as shown in Fig 3, the population with 100% re-identification risk increases very fast until the number of spatio-temporal points reaches four. Nearly **75%** of the population can be uniquely identified by four spatio-temporal points. The re-identification risk then shows a convergent trend with a maximum risk of nearly **80%.**

As reported in [16], four spatio-temporal points are enough to identify uniquely 95% of mobile users in a small European country. Study [16] also suggested that their results should generalize to higher population densities based on the following considerations. On the one hand, higher population density tends to increase the anonymity set; on the other hand, more mobile base stations will locate in areas with high population density, thus increasing the spatial precision of trajectories, which should offset the influence of high population density. However, the results of this research suggest significantly lower privacy risks in Shenzhen City, an area very likely having a much higher population density than the small European country. According to the results presented here, more mobile base stations as a result of higher population density might not fully offset the influence of high population density. However, the distributions of mobile base stations and population density used in study [16] are not available, and therefore the offsetting effect of base station density against population density cannot be evaluated. Moreover, as shown in Table 3, the re-identification risks in various cities in the United States increase with population density. Therefore, according to the results of this study and of studies [15–16], besides population density, factors such as degree of urban lifestyle must be considered to explain the spatial heterogeneity of re-identification risks.

### Re-Identification Risk Reduction after Privacy Protection

**Fig 4:**
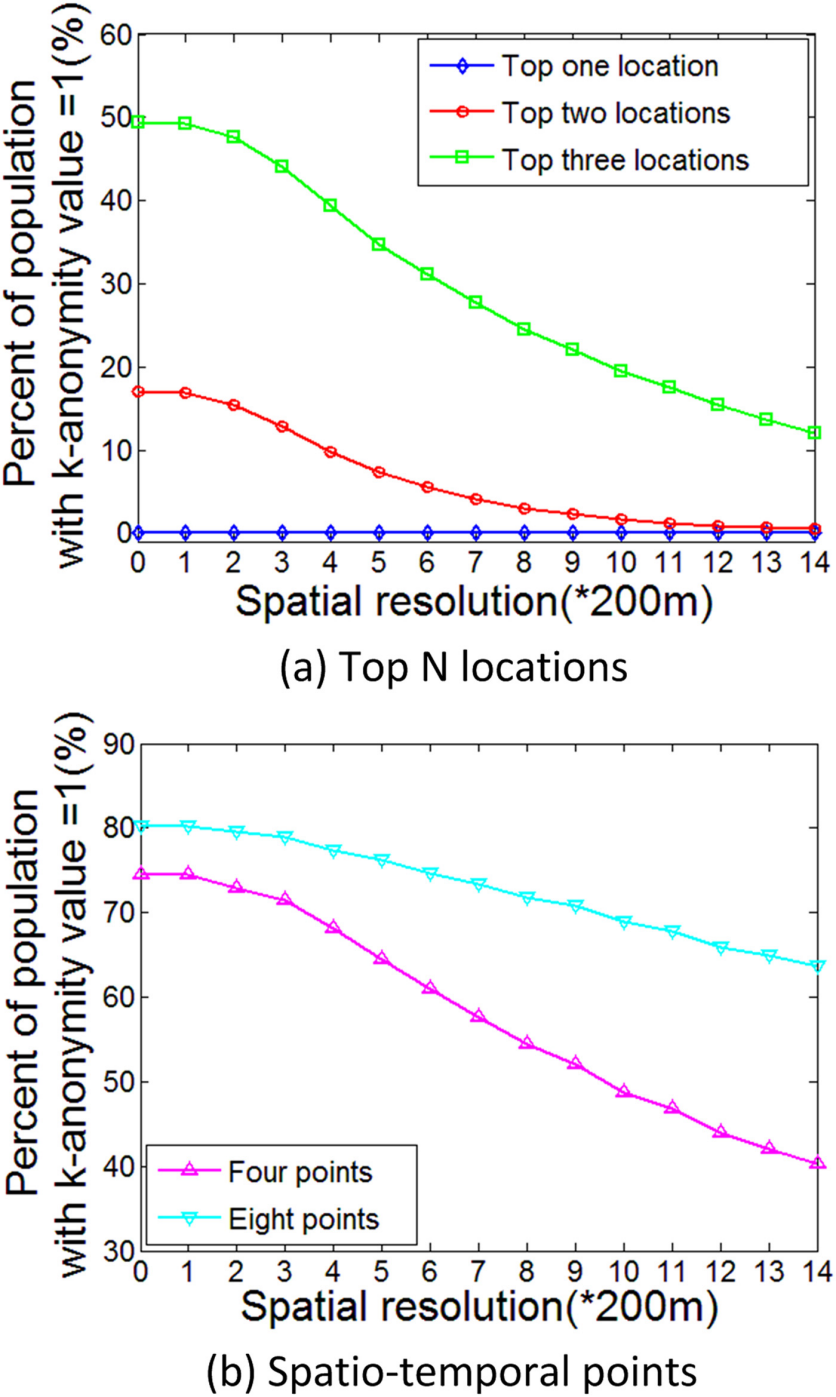
Privacy risk reduction of two privacy models. (a) Top N locations. (b) Spatio-temporal points.

The spatial generalization approach proposed by this study can effectively reduce the re-identification risks for the top two and three locations, and the re-identification risk declines more sharply for the top three locations (Fig 4a). When spatial aggregation scale increases to 2800 m, the uniquely identified population drops from 17% to 1% for the top two locations and from 49% to 12% for the top three locations.

As for the attack model using spatio-temporal points, to illustrate the effect of the spatial generalization approach on reducing re-identification risk, four and eight spatio-temporal points were chosen, because they are two critical points on the curve shown in Fig 3. When the spatial aggregation scale increased to 2800 m, the uniquely identified population dropped from 75% to 40% when using four spatio-temporal points and from 80% to 64% when using eight spatio-temporal points. The slower rate of decrease when using eight spatio-temporal points indicates the increased difficulty of reducing re-identification risk by spatial generalization when attackers know more target footprints.

### Data Utility Loss after Privacy Protection

**Fig 5:**
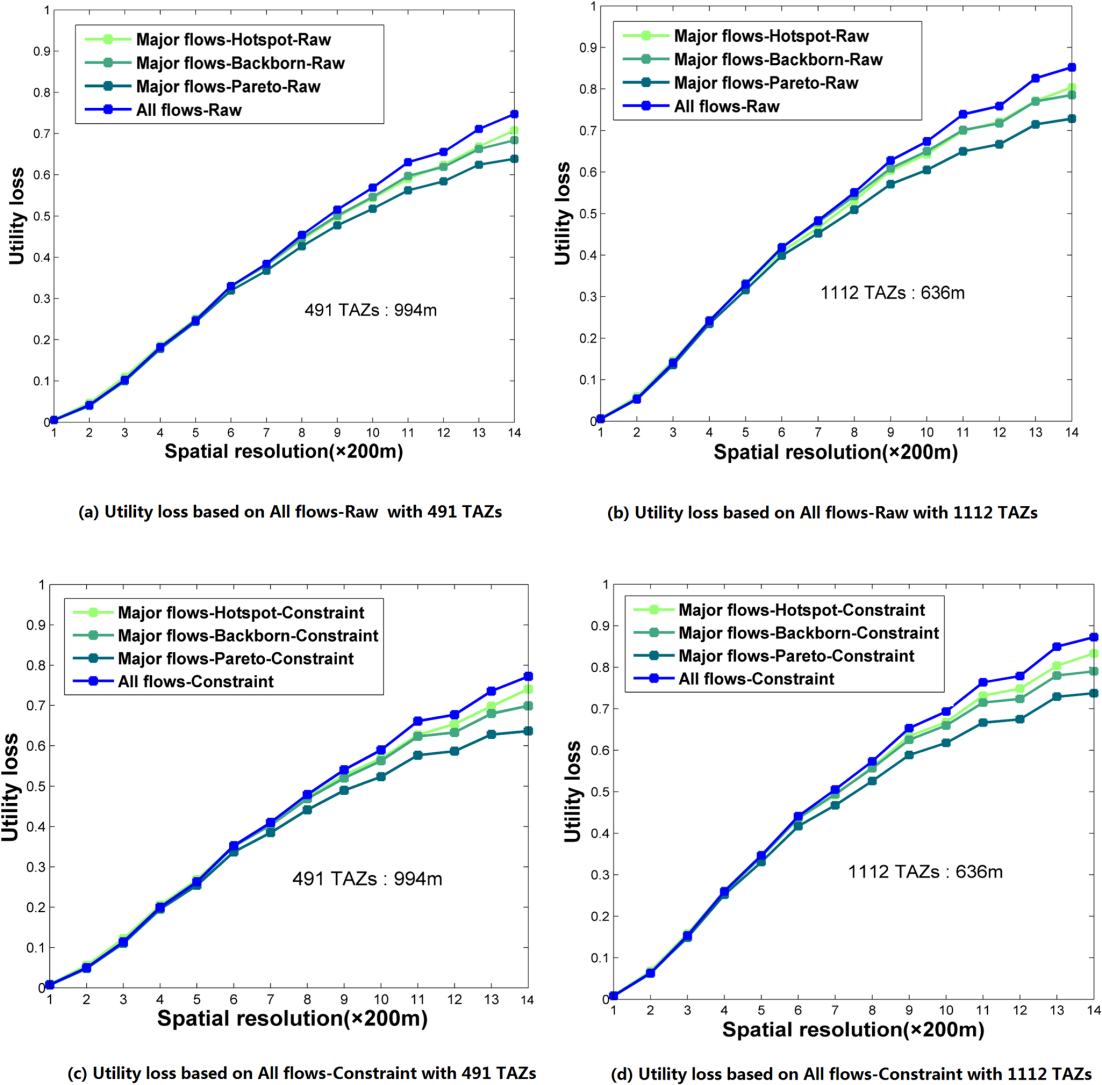
Data utility loss on analyzing OD flows with spatial generalization resolution. (a) Utility loss based on All flows-Raw with 491 TAZs. (b) Utility loss based on All flows-Raw with 1112 TAZs. (c) Utility loss based on All flows-Constraint with 491 TAZs. (d) Utility loss based on All flows-Constraint with 1112 TAZs.

**Fig 6:**
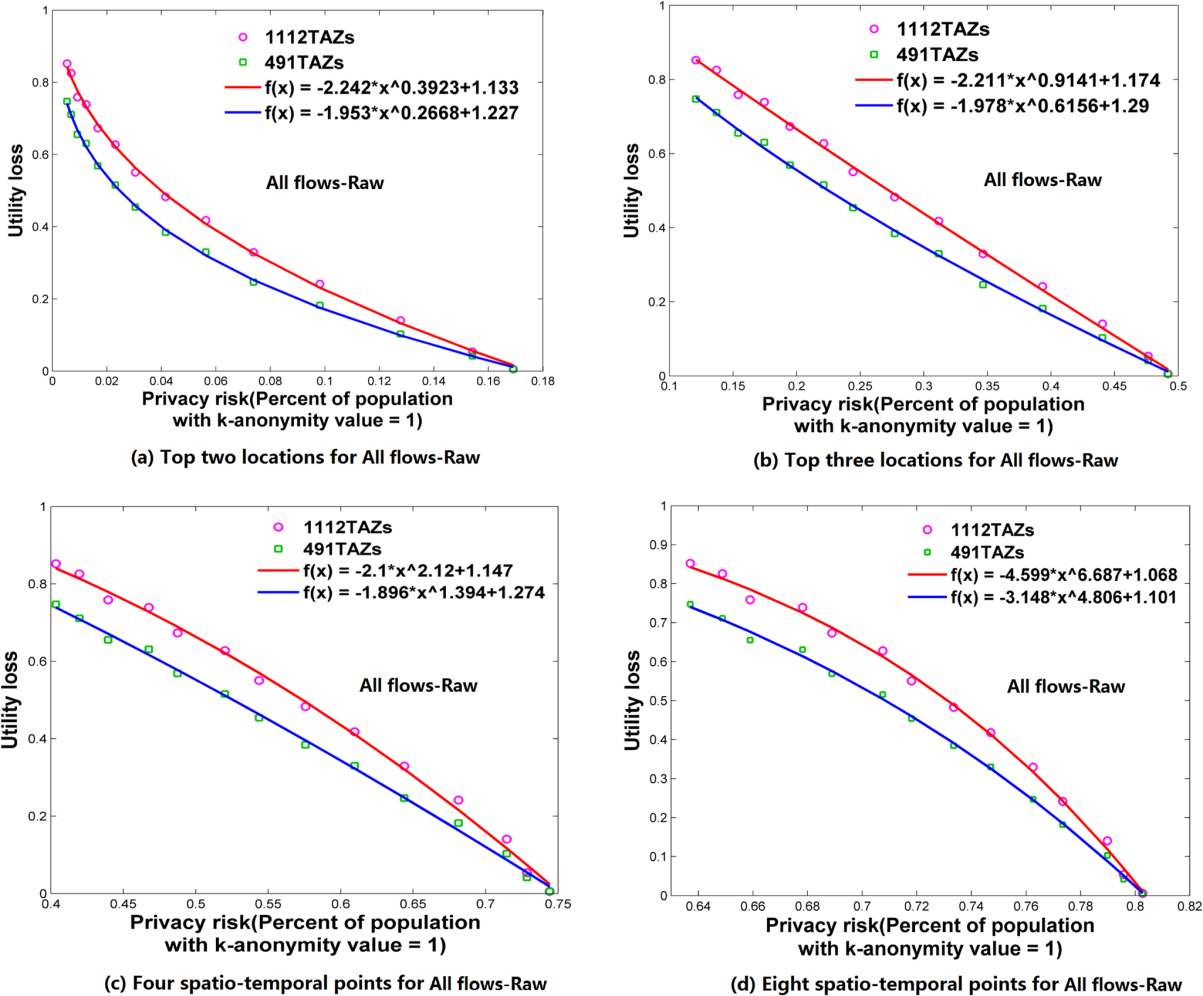
Relationships between privacy risk and data utility using All flows-Raw. (a) Top two locations. (b) Top three locations. (c) Four spatio-temporal points. (d) Eight spatio-temporal points.

**Fig 7:**
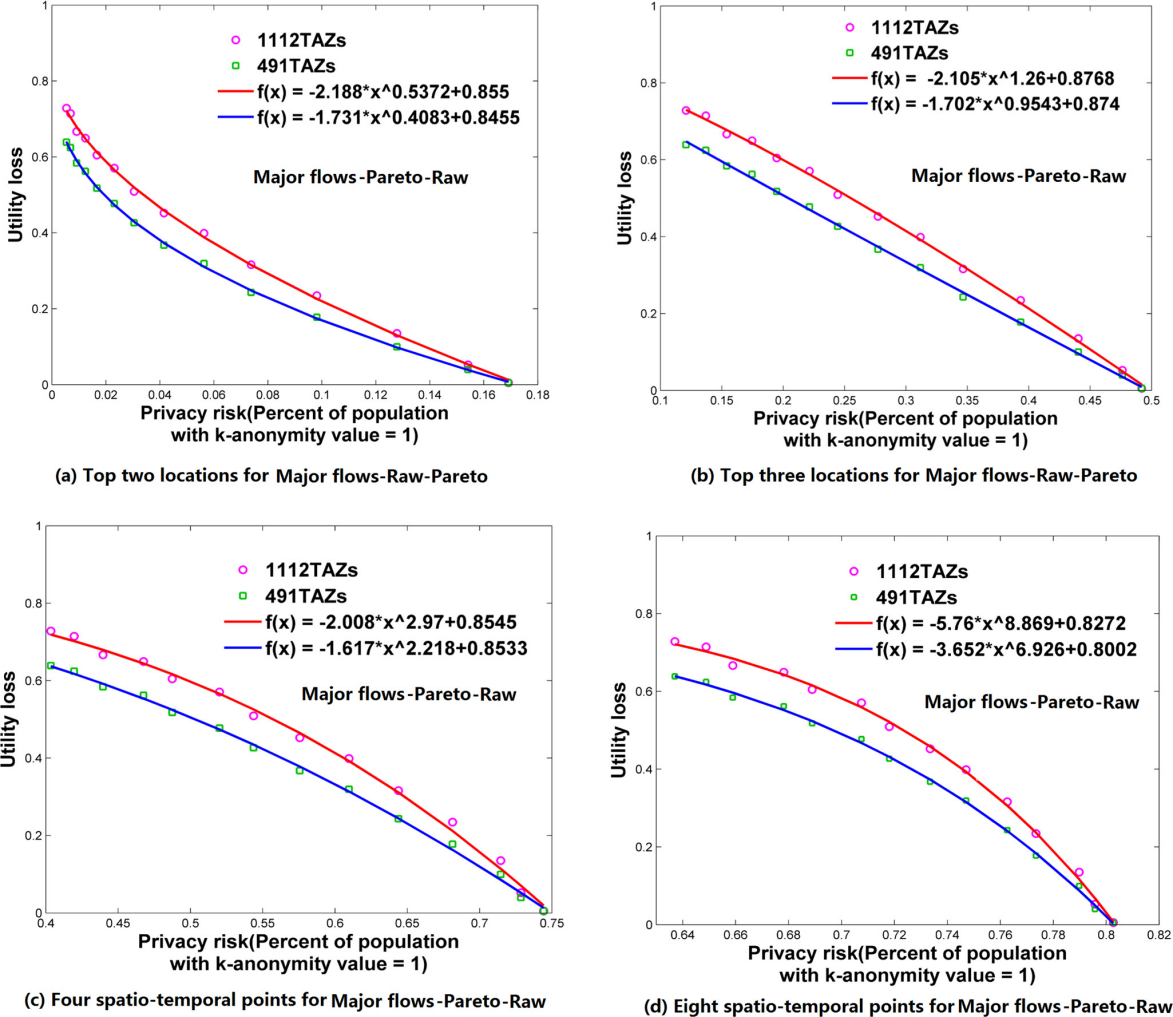
Relationships between privacy risk and data utility using Major flows-Pareto-Raw. (a) Top two locations. (b) Top three locations. (c) Four spatio-temporal points. (d) Eight spatio-temporal points.

**Fig 8:**
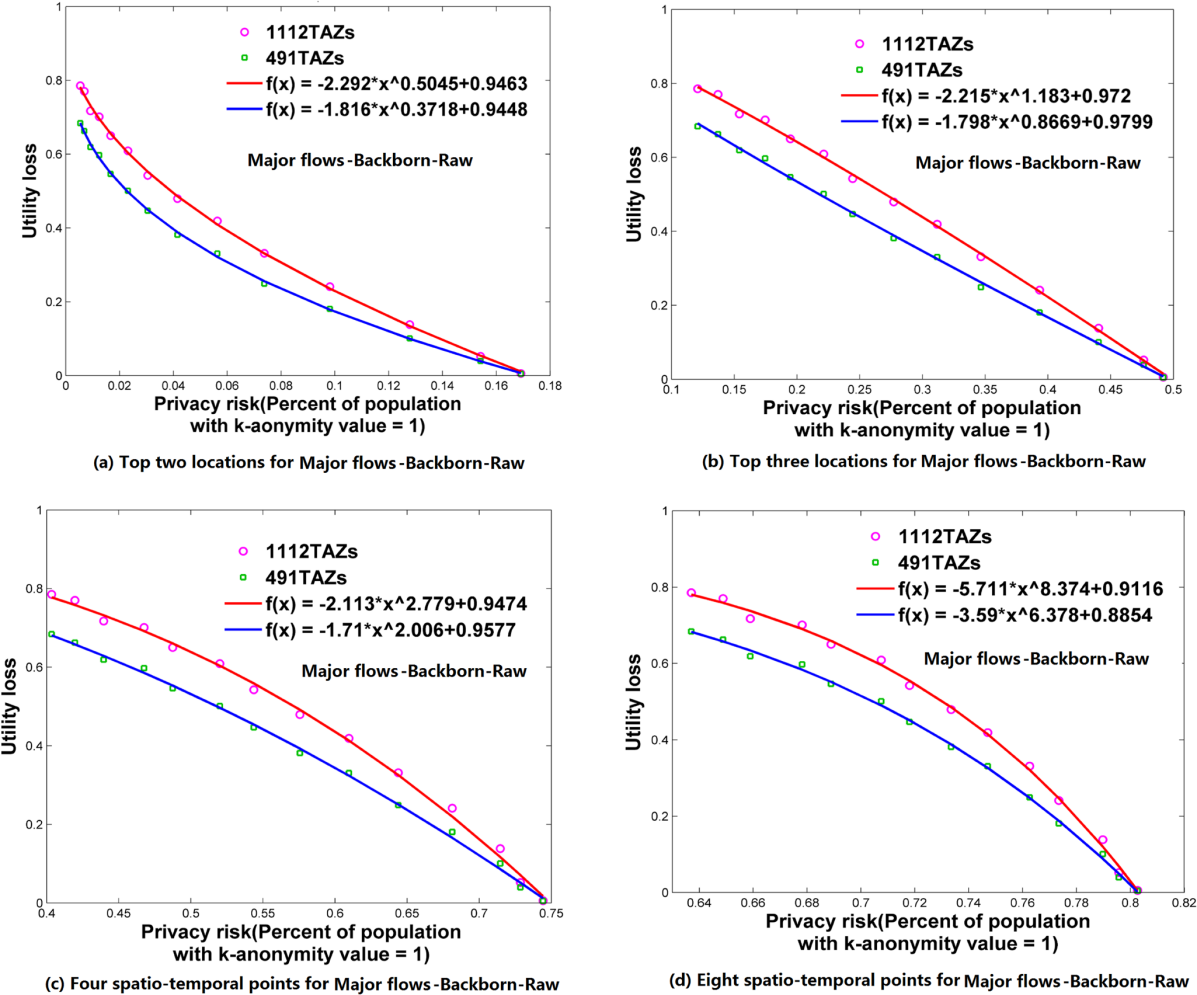
Relationships between privacy risk and data utility using Major flows-Backborn-Raw. (a) Top two locations. (b) Top three locations. (c) Four spatio-temporal points. (d) Eight spatio-temporal points.

**Fig 9:**
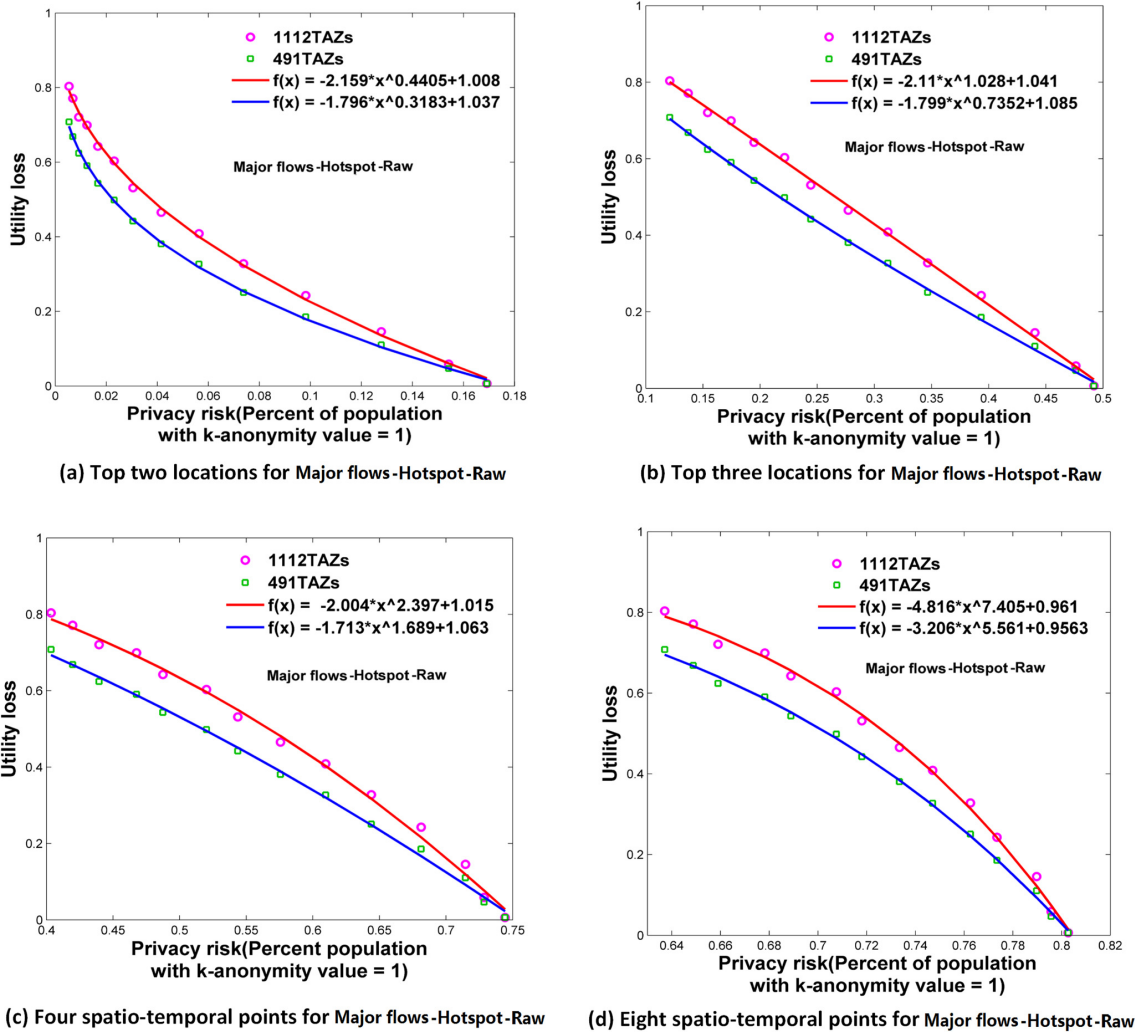
Relationships between privacy risk and data utility using Major flows-Hotspot-Raw. (a) Top two locations. (b) Top three locations. (c) Four spatio-temporal points. (d) Eight spatio-temporal points.

**Fig 10:**
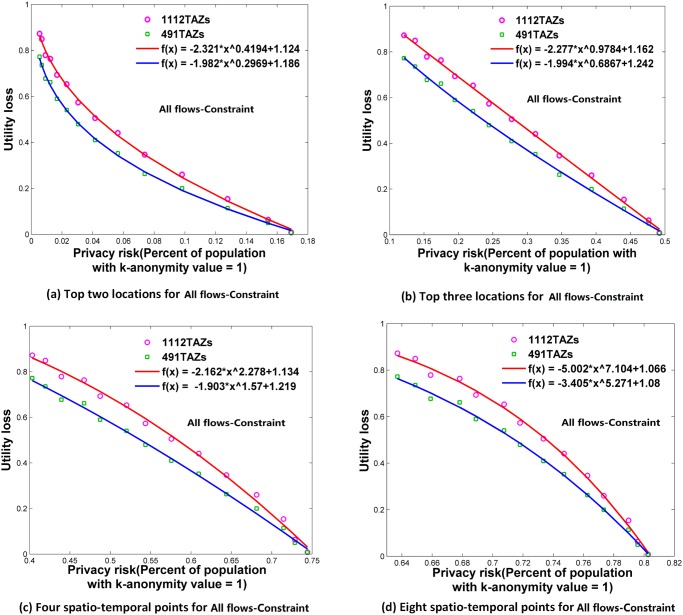
Relationships between privacy risk and data utility using All flows-Constraint. (a) Top two locations. (b) Top three locations. (c) Four spatio-temporal points. (d) Eight spatio-temporal points.

**Fig 11:**
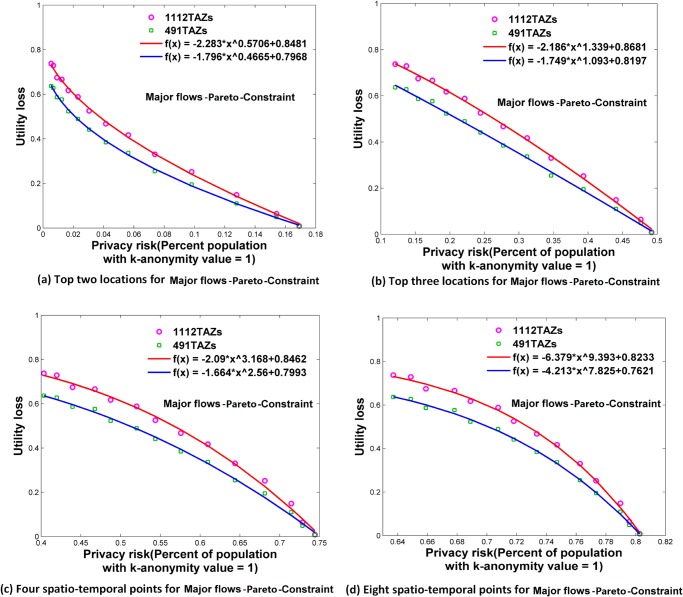
Relationships between privacy risk and data utility using Major flows-Pareto-Constraint. (a) Top two locations. (b) Top three locations. (c) Four spatio-temporal points. (d) Eight spatio-temporal points.

**Fig 12:**
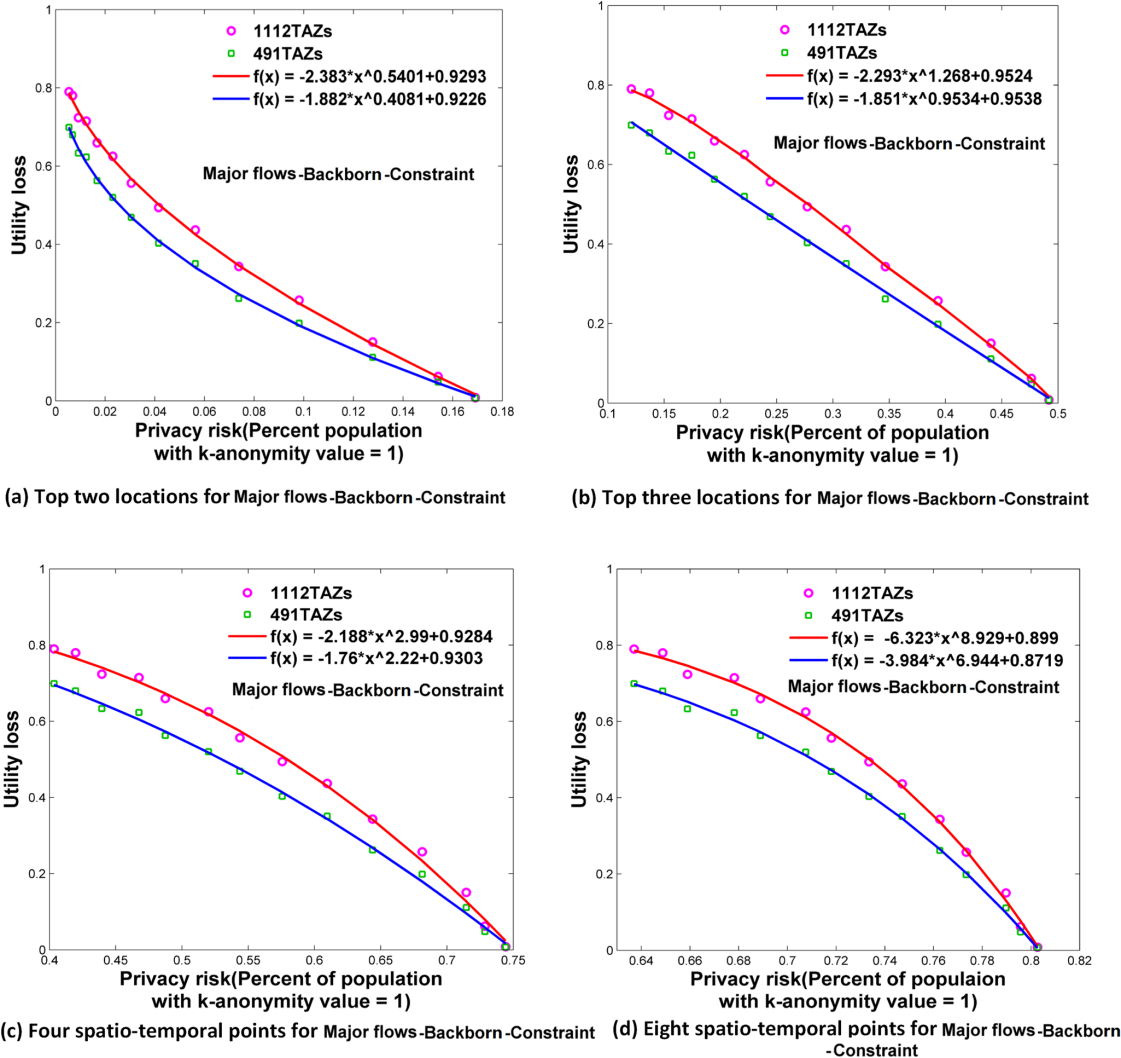
Relationships between privacy risk and data utility using Major flows-Backborn-Constraint. (a) Top two locations. (b) Top three locations. (c) Four spatio-temporal points. (d) Eight spatio-temporal points.

**Fig 13:**
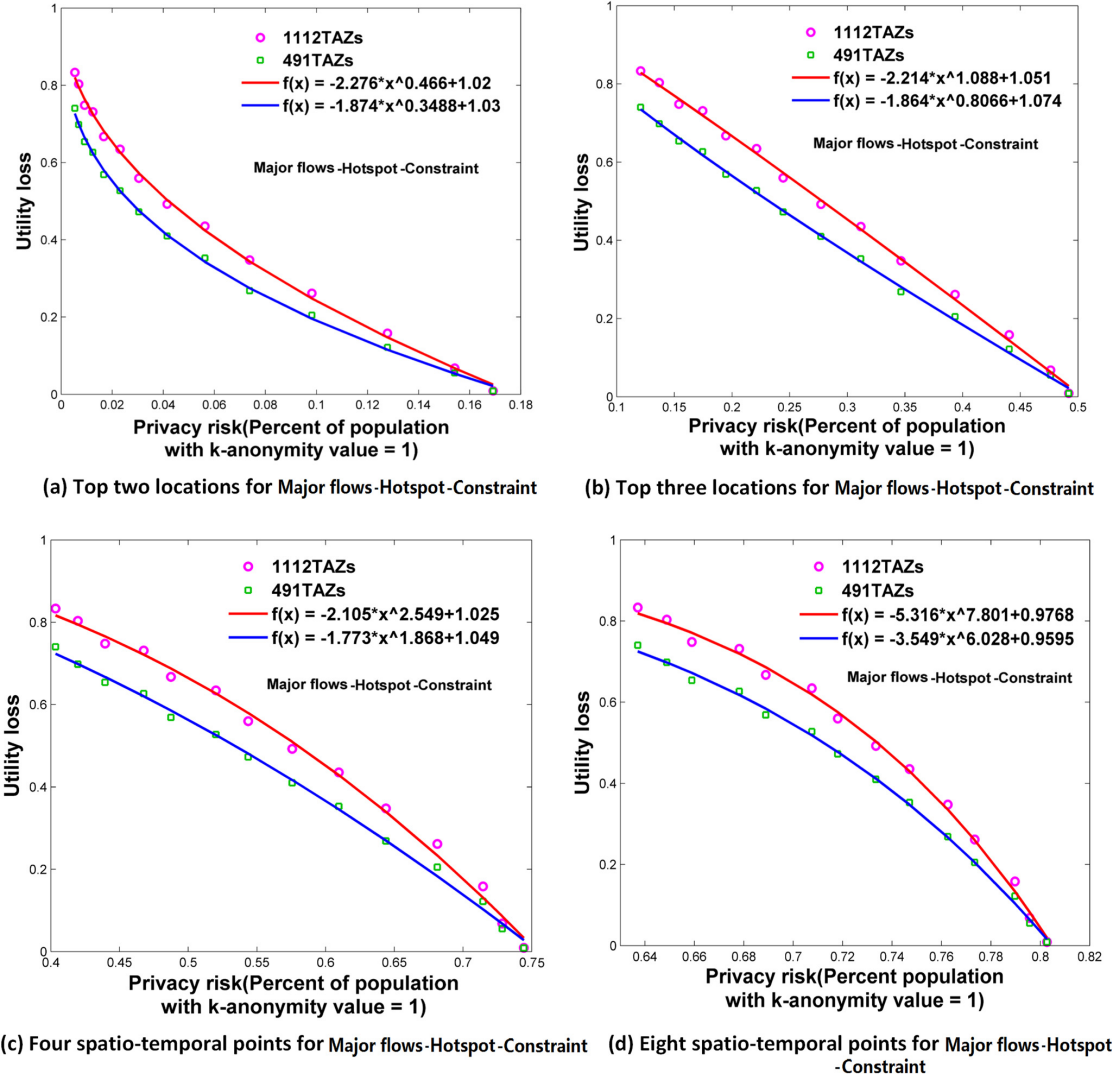
Relationships between privacy risk and data utility using Major flows-Hotspot-Constraint. (a) Top two locations. (b) Top three locations. (c) Four spatio-temporal points. (d) Eight spatio-temporal points.

Using OD flow analysis as a data utility, Fig 5 shows that when spatial generalization scale increases from 200 m to 2800 m, data utility loss significantly increases while the differences between various data utility measurements gradually expand. When the spatial generalization scale increases to larger than 1000 m, the impact of privacy protection on major flows becomes slightly less than that of all flows. Because of the significant importance of major flows in transportation systems, the authors suggest using the major flow curves as a more important reference in practice. Besides, the data utility loss in an analysis based on TAZs with larger spatial units (491 TAZs with an average radius of 994 m) is clearly less than that in an analysis based on TAZs with smaller spatial units (1112 TAZs with an average radius of 636 m). This pattern can also be found in Figs 6–13 in the next section.

### Relationships between Re-Identification Risk and Data Utility

Using the attack models of top *N* locations and spatio-temporal points, Figs 6–9 and 10–13 present the relationships between re-identification risk and data utility loss respectively based on the original OD flows (i.e. the ‘-Raw’ series) and the OD flows with 5~100 min time constraints (i.e. the ‘-Constraint’ series). It was found that all the relationships between re-identification risk ***x*** (the percentage of the population with 100% re-identification risk) and data utility loss ***y*** for the two attack models can be expressed by one formula: *y = -ax* ^*b*^ *+c (a*,*b*,*c>0;0<x<1)*. All estimators for *a*, *b*, and *c* are statistically significant (*p*<0.001), and the values of *R* ^2^ are more than 0.99 for all cases (see S2 Table), which indicate the goodness of fit of the proposed function.

The exponent *b* determines the shape of the curves. When *b* is between 0 and 1, such as for the top two locations (Fig 6a), to diminish the re-identification risk (please look at the *x* axis from right to left), the cost in data utility of reducing the re-identification risk is relatively low at the beginning, but gradually increases. When *b* is close to 1, such as for the top three locations (the red curve in Fig 6b for 1112 TAZs), the tradeoff between re-identification risk and data utility loss is close to linear. When *b* is larger than 1, such as for four and eight spatio-temporal points (Fig 6c and 6d), the cost in data utility for reducing privacy is relatively high at the beginning and gradually becomes lower. In general, a smaller *b* implies a larger marginal utility of the tradeoff for data utility, thus providing an easier way to protect privacy in the beginning phase that is more helpful in practice.

As shown in Figs 6–13, either for all flows or for major flows, the exponent *b* increases from the top two locations to the top three locations, to four spatio-temporal points, and then to eight spatio-temporal points. This trend indicates an increase in background knowledge of attackers in these four models and accordingly the increasing difficulty of protecting privacy. The uniform relationship between re-identification risk and data utility for the two attack models also offers a way to compare the two attack models. For example, the slightly larger *b* in the four spatio-temporal points model than in the top three locations model indicates a slight increase in the background knowledge of attackers as well as an increase in the difficulty of protecting privacy.

This study designed five different data utility measurements for OD flow analysis, including the raw OD flows, OD flows with time constraints, and three types of major flows. If the raw OD flows form a complete set, the others are four types of subsets with different filtering methods. In terms of the relationship between re-identification risk and data utility loss, our results show that subsets of OD flows created by a filtering method have the same relationship with the complete set of OD flows, and the filtering method does not affect the relationship.

### Contributions and Future Work

The contributions of this research include the following two aspects. First, this study strengthens the argument that spatial heterogeneity exists in the re-identification risks in call detail records. Study [15] has demonstrated the spatial heterogeneity of different American cities under the attack model using the top *N* locations. Study [16] did not address this issue, but it inferred a spatial homogeneity under the attack model using spatial-temporal points. The results of this research clearly show that the re-identification risks of Shenzhen’s mobile phone users lie between those of Sacramento and those of Chicago and are significantly lower than those in the reported European country when using spatial-temporal points. This study has therefore demonstrated that spatial heterogeneity not only occurs in the attack model using the top *N* locations, but is also valid in the attack model using spatial-temporal points. This finding raises awareness that the re-identification risks in call detail records vary across regions, and therefore that methods and institutions of protecting privacy when sharing detailed individual trajectory data should be considered in a local context. Second, a uniform relationship was found between re-identification risks (*x*) and the selected data utility (*y*) for both the top *N* location model and the spatio-temporal point model: *y = -ax* ^*b*^ *+c (a*,*b*,*c>0;0<x<1)*where the exponent *b* increases with the background knowledge of the attackers and a larger *b* implies more difficulty in reducing re-identification risk in the early phase. This relationship offers a tradeoff reference for other aggregated analyses based on mobile phone location data. It also provides data publishers with useful guidance on choosing the right tradeoff between privacy and utility.

This study raises several issues that need to be addressed in future work. (1) Based on the findings of this research and on previous studies [15–16], the spatial heterogeneity of the re-identification risks in call detail records likely comes from many reasons such as phone usage habits, population density, and lifestyle. To obtain an in-depth understanding of the factors influencing re-identification risks, it is necessary to investigate the quantitative relationship between privacy risk in mobile traces and possible influencing factors. (2) The proposed spatial generalization method is able to reduce re-identification risks in mobile traces. However, the data utility of generating an OD flow matrix, which is a typical aggregated mobility analysis, decreases rapidly when this procedure is applied to the dataset. Therefore, efforts are still needed to improve privacy protection methods for mobile phone location datasets. (3) Besides aggregated mobility analysis, the impact of privacy protection on analyses at an individual level requires further study. (4) If data is available in future, a study comparing with other regions would be worth pursuing.

## Supporting Information

S1 Fig Aggregated base stations by a series of grids ranging from 200 m × 200 m to 2800 m × 2800 m with an increment of 200 m. (TIF)

S2 Fig The travel time distribution of a single trip in the travel dairy of Shenzhen City (sample size = 190,000). (TIF)

S3 Fig The cumulative distribution of flows based on two sets of TAZs. (a) 491 TAZs. (b) 1112 TAZs.
(TIF)

S1 Table A snapshot of the CDR dataset. (DOCX)

S2 Table The goodness of fit of the proposed function *(y = -ax* ^*b*^ *+c)*
.
(DOCX)
