# Supplementary figures and images for: Re-Identification Risk versus Data Utility for Aggregated Mobility Research Using Mobile Phone Location Data
Source: PLoS One. 2015 Oct 15;10(10):e0140589. doi: 10.1371/journal.pone.0140589 (PMC4607417; doi:10.1371/journal.pone.0140589)

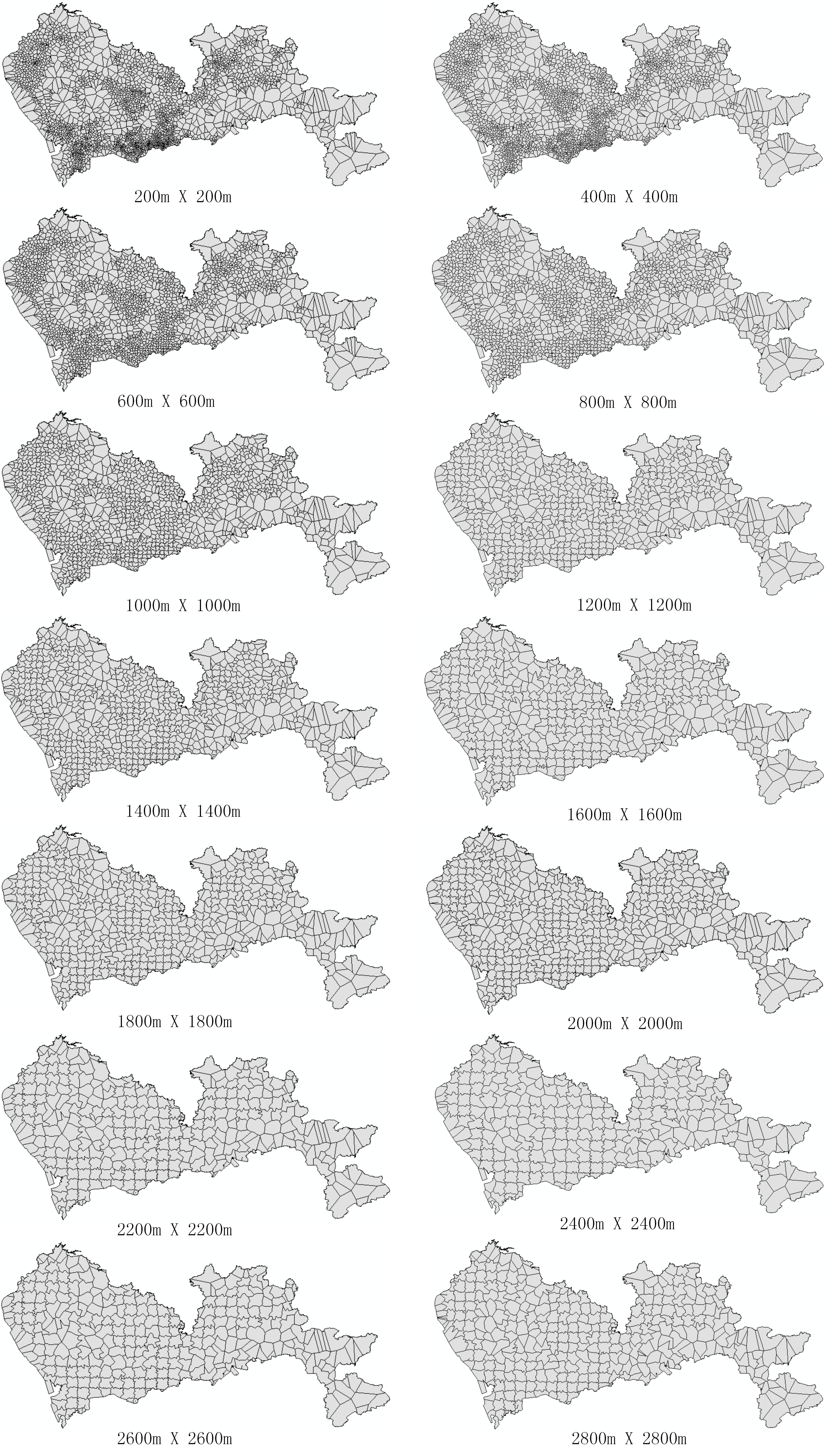

Supplement: S1 Fig — (TIF) [file pone.0140589.s001.tif]

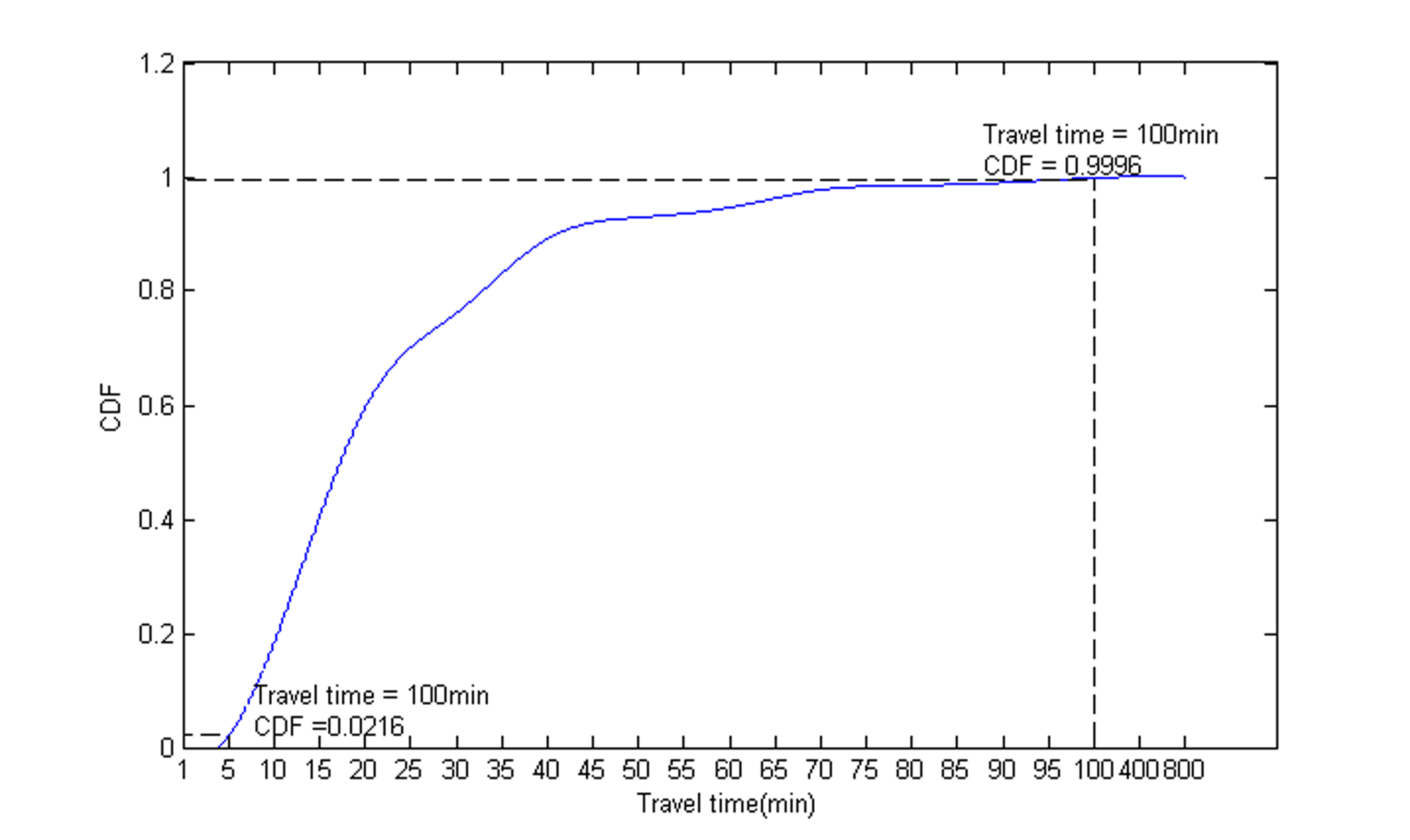

Supplement: S2 Fig — (TIF) [file pone.0140589.s002.tif]

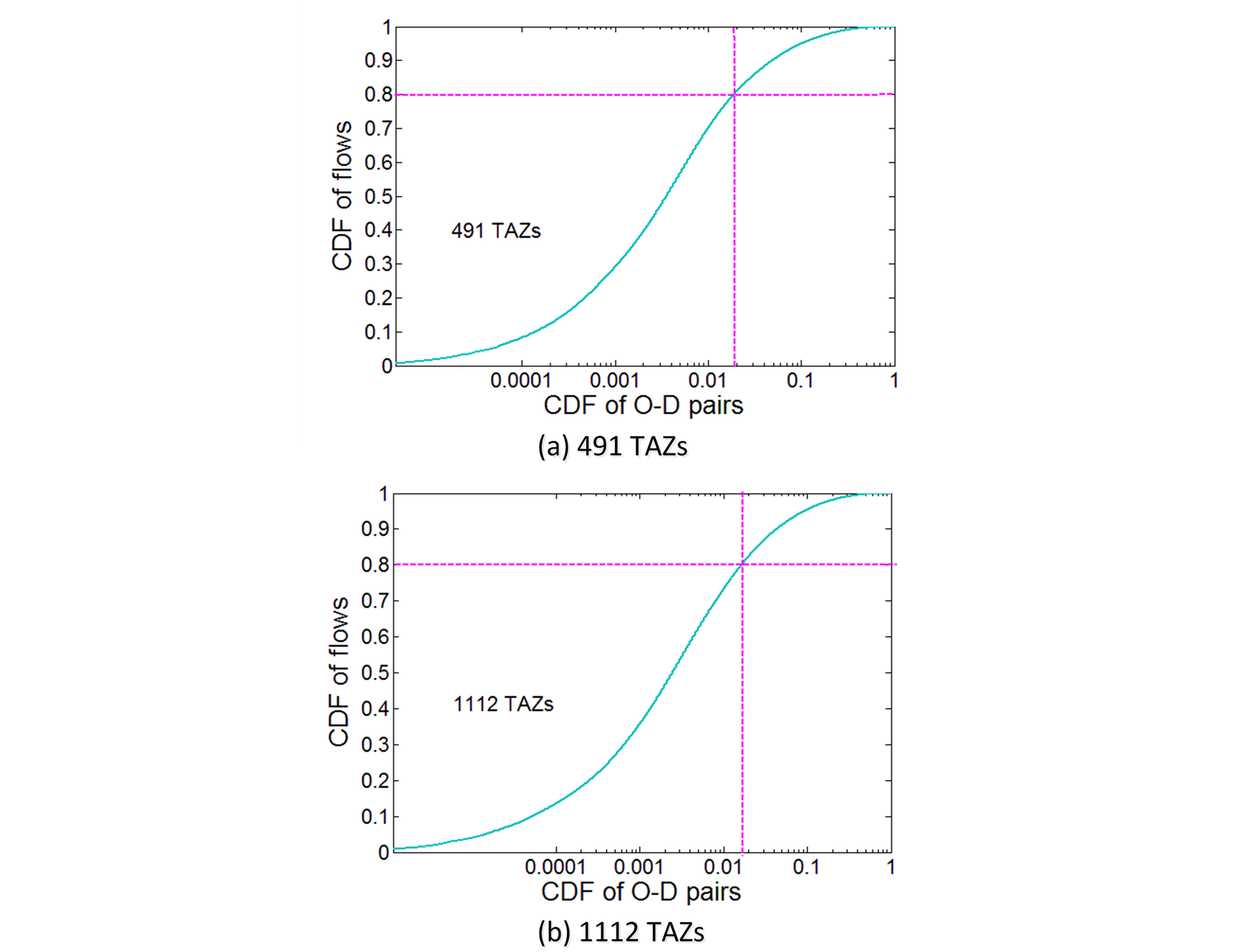

Supplement: S3 Fig — (a) 491 TAZs. (b) 1112 TAZs. (TIF) [file pone.0140589.s003.tif]
