# Supplementary material for: Re-Identification Risk versus Data Utility for Aggregated Mobility Research Using Mobile Phone Location Data
Source: PLoS One. 2015 Oct 15;10(10):e0140589. doi: 10.1371/journal.pone.0140589 (PMC4607417; doi:10.1371/journal.pone.0140589)
Supplement: S1 Table — (DOCX) [file pone.0140589.s004.docx]

**S1 Table. A snapshot of the CDR dataset**

| ID | Time | Latitude | Longitude | Flag | Regional code |
| --- | --- | --- | --- | --- | --- |
| 000****850 | 201*/*/1 6:03 | 22.542534 | 114.11719 | 1 | 755 |
| 000****850 | 201*/*/1 9:42 | 22.542534 | 114.11719 | 1 | 755 |
| 000****850 | 201*/*/1 10:27 | 22.542534 | 114.11719 | 0 | 755 |
| 000****850 | 201*/*/1 15:38 | 22.542534 | 114.11719 | 0 | 755 |
| 000****850 | 201*/*/1 16:06 | 22.542534 | 114.11719 | 1 | 755 |
| 000****850 | 201*/*/1 16:15 | 22.544722 | 114.119444 | 0 | 755 |
| 000****850 | 201*/*/1 16:56 | 22.546667 | 114.120306 | 1 | 755 |
| 000****850 | 201*/*/1 18:00 | 22.550833 | 114.122222 | 1 | 755 |
| 000****850 | 201*/*/1 19:46 | 22.548929 | 114.111791 | 1 | 755 |
| 000****850 | 201*/*/1 20:22 | 22.546667 | 114.120306 | 0 | 755 |
| 000****850 | 201*/*/1 20:24 | 22.546667 | 114.120306 | 0 | 755 |
| 000****850 | 201*/*/1 20:47 | 22.546667 | 114.120306 | 0 | 755 |
| 000****850 | 201*/*/1 21:15 | 22.546667 | 114.120306 | 0 | 755 |
| 000****850 | 201*/*/1 21:34 | 22.546667 | 114.120306 | 0 | 755 |
| 000****850 | 201*/*/1 22:00 | 22.546667 | 114.120306 | 1 | 755 |
| 000****850 | 201*/*/1 22:24 | 22.55195 | 114.12525 | 0 | 755 |
| 000****850 | 201*/*/1 22:52 | 22.55195 | 114.12525 | 1 | 755 |
| 000****850 | 201*/*/1 22:53 | 22.55195 | 114.12525 | 1 | 755 |
| 000****850 | 201*/*/1 23:12 | 22.550833 | 114.125833 | 0 | 755 |

S1 Table demonstrates the call detail records of user 000****850 (encrypted) on day 201*/*/1.
