# Supplementary material for: Re-Identification Risk versus Data Utility for Aggregated Mobility Research Using Mobile Phone Location Data
Source: PLoS One. 2015 Oct 15;10(10):e0140589. doi: 10.1371/journal.pone.0140589 (PMC4607417; doi:10.1371/journal.pone.0140589)
Supplement: S2 Table — . (DOCX) [file pone.0140589.s005.docx]

**S2 Table. The goodness of fit of the proposed function (*y = -ax^b^+c*)**

| Case | Value of R-square |
| --- | --- |
| Based on All flows-Raw:  Top two locations for All flows with 1112 TAZs | 0.9988 |
| Top two locations for All flows with 491 TAZs | 0.9993 |
| Top two locations for Major flows-Pareto with 1112 TAZs | 0.9990 |
| Top two locations for Major flows- Pareto with 491 TAZs | 0.9996 |
| Top two locations for Major flows-Backborn with 1112 TAZs | 0.9993 |
| Top two locations for Major flows-Backborn with 491 TAZs  Top two locations for Major flows-Hotspot with 1112 TAZs  Top two locations for Major flows-Hotspot with 491 TAZs  Top three locations for All flows with 1112 TAZs | 0.9995  0.9986  0.9992  0.9987 |
| Top three locations for All flows with 491 TAZs | 0.9990 |
| Top three locations for Major flows-Pareto with 1112 TAZs | 0.9990 |
| Top three locations for Major flows-Pareto with 491 TAZs | 0.9991 |
| Top three locations for Major flows-Backborn with 1112 TAZs | 0.9991 |
| Top three locations for Major flows-Backborn with 491 TAZs | 0.9991 |
| Top three locations for Major flows-Hotspot with 1112 TAZs  Top three locations for Major flows-Hotspot with 491 TAZs | 0.9986  0.9992 |
| Four spatio-temporal points for All flows with 1112 TAZs | 0.9965 |
| Four spatio-temporal points for All flows with 491 TAZs | 0.9978 |
| Four spatio-temporal points for Major flows-Pareto with 1112 TAZs | 0.9962 |
| Four spatio-temporal points for Major flows-Pareto with 491 TAZs | 0.9983 |
| Four spatio-temporal points for Major flows-Backborn with 1112 TAZs | 0.9973 |
| Four spatio-temporal points for Major flows- Backborn with 491 TAZs  Four spatio-temporal points for Major flows-Hotspot with 1112 TAZs  Four spatio-temporal points for Major flows-Hotspot with 491 TAZs  Eight spatio-temporal points for All flows with 1112 TAZs | 0.9983  0.9960  0.9975  0.9974 |
| Eight spatio-temporal points for All flows with 491 TAZs | 0.9983 |
| Eight spatio-temporal points for Major flows-Pareto with 1112 TAZs | 0.9978 |
| Eight spatio-temporal points for Major flows-Pareto with 491 TAZs | 0.9987 |
| Eight spatio-temporal points for Major flows-Backborn with 1112 TAZs | 0.9980 |
| Eight spatio-temporal points for Major flows- Backborn with 491 TAZs | 0.9986 |
| Eight spatio-temporal points for Major flows-Hotspot with 1112 TAZs  Eight spatio-temporal points for Major flows-Hotspot with 491 TAZs | 0.9973  0.9984 |

| Case | Value of R-square |
| --- | --- |
| Based on All flows-Constraint:  Top two locations for All flows with 1112 TAZs | 0.9984 |
| Top two locations for All flows with 491 TAZs | 0.9989 |
| Top two locations for Major flows-Pareto with 1112 TAZs | 0.9984 |
| Top two locations for Major flows- Pareto with 491 TAZs | 0.9990 |
| Top two locations for Major flows-Backborn with 1112 TAZs | 0.9988 |
| Top two locations for Major flows-Backborn with 491 TAZs  Top two locations for Major flows-Hotspot with 1112 TAZs  Top two locations for Major flows-Hotspot with 491 TAZs  Top three locations for All flows with 1112 TAZs | 0.9990  0.9982  0.9988  0.9983 |
| Top three locations for All flows with 491 TAZs | 0.9985 |
| Top three locations for Major flows-Pareto with 1112 TAZs | 0.9982 |
| Top three locations for Major flows-Pareto with 491 TAZs | 0.9984 |
| Top three locations for Major flows-Backborn with 1112 TAZs | 0.9985 |
| Top three locations for Major flows-Backborn with 491 TAZs | 0.9985 |
| Top three locations for Major flows-Hotspot with 1112 TAZs  Top three locations for Major flows-Hotspot with 491 TAZs | 0.9982  0.9989 |
| Four spatio-temporal points for All flows with 1112 TAZs | 0.9959 |
| Four spatio-temporal points for All flows with 491 TAZs | 0.9972 |
| Four spatio-temporal points for Major flows-Pareto with 1112 TAZs | 0.9959 |
| Four spatio-temporal points for Major flows-Pareto with 491 TAZs | 0.9974 |
| Four spatio-temporal points for Major flows-Backborn with 1112 TAZs | 0.9966 |
| Four spatio-temporal points for Major flows- Backborn with 491 TAZs  Four spatio-temporal points for Major flows-Hotspot with 1112 TAZs  Four spatio-temporal points for Major flows-Hotspot with 491 TAZs  Eight spatio-temporal points for All flows with 1112 TAZs | 0.9977  0.9955  0.9970  0.9971 |
| Eight spatio-temporal points for All flows with 491 TAZs | 0.9979 |
| Eight spatio-temporal points for Major flows-Pareto with 1112 TAZs | 0.9973 |
| Eight spatio-temporal points for Major flows-Pareto with 491 TAZs | 0.9983 |
| Eight spatio-temporal points for Major flows-Backborn with 1112 TAZs | 0.9976 |
| Eight spatio-temporal points for Major flows- Backborn with 491 TAZs | 0.9983 |
| Eight spatio-temporal points for Major flows-Hotspot with 1112 TAZs  Eight spatio-temporal points for Major flows-Hotspot with 491 TAZs | 0.9969  0.9981 |
